# Multicorrection Goldenhar syndrome (facio-auriculo-vertebral dysplasia): a rare follow-up case of 12-year-old female

**DOI:** 10.11604/pamj.2021.39.96.27259

**Published:** 2021-06-02

**Authors:** Ashish Ramesh Varma, Revat Jagdish Meshram, Anuj Ramesh Varma, Anubhuti Sunil Dixit, Siddhart Sunil Zabak, Chaitanya Ajay Kulkarni

**Affiliations:** 1 Department of Paediatrics, Jawaharlal Nehru Medical College, Datta Meghe Institute of Medical Sciences (Deemed to be University), Ravi Nair College of Physiotherapy, Sawangi (M), Wardha-442001, Maharashtra, India,; 2 Department of Medicine, Jawaharlal Nehru Medical College, Datta Meghe Institute of Medical Sciences (Deemed to be University), Ravi Nair College of Physiotherapy, Sawangi (M), Wardha-442001, Maharashtra, India,; 3 Department of Community Physiotherapy, Jawaharlal Nehru Medical College, Datta Meghe Institute of Medical Sciences (Deemed to be University), Ravi Nair College of Physiotherapy, Sawangi (M), Wardha-442001, Maharashtra, India

**Keywords:** Goldenhar syndrome, female, facio-auriculo-vertebral dysplasia, cardiac syndromes, case report

## Abstract

The MSX homeobox genes cause Goldenhar syndrome (GHS) or facio-auriculo-vertebral dysplasia, a rare developmental defect. Its exact etiology is still unknown. Its incidence lies between 1: 3500 and 1: 5600. In 85% of the cases, the unilateral face is affected. Typical clinical findings in a classic GHS include eye disorders, ear irregularities (with or without hearing loss), facial impairments, dental and oral ailments, cardiac syndromes, central nervous system (CNS) involvement, trachea and lung malformations, kidney and gastrointestinal defects, and skeletal alterations. This case report presents a follow-up case of Goldenhar Syndrome in a 12-year-old female, with no relevant family history, diagnosed with anotia on the left side, cyanosis, and facial asymmetry at birth. She presented with moderate growth failure, bilateral sclerosing mastoiditis and kyphoscoliosis. She underwent posterior scoliosis correction posterior instrumented fusion from D1 to D11.

## Introduction

Goldenhar syndrome or facio-auriculo-vertebral dysplasia, a rare developmental defect, was first described by Carl Ferdinand Von Arlt in 1981 [1,2]. Goldenhar, in 1952, classified its clinical variations [2,3]. Its pathogenesis is caused by the MSX homeobox genes. Its incidence lies between 1: 3500 and 1: 5600, wherein the male to female ratio is 3: 2 [1,4]. In 85% of GHS cases, the unilateral face is affected, the right side being commonly involved [2,3]. Rarely, there is bilateral involvement. Its exact etiology is still unknown. Nevertheless, the etiology of GHS can be due to exposure to multiple viruses such as rubella and influenza, or prescription drugs during pregnancy. Besides, maternal diabetes can also be the causative factor [3,5]. Since GHS involves first and second bronchial arches, it is categorized into a first and second brachial arch syndrome, respectively [1,3]. Typical clinical findings in a classic GHS include eye disorders, ear irregularities (with or without hearing loss), facial impairments, dental and oral ailments, cardiac syndromes, central nervous system (CNS) involvement, trachea and lung malformations, kidney and gastrointestinal defects, and skeletal alterations [1,5]. Laboratory, radiological, and clinical findings form the basis for the diagnosis of GHS [3]. This case report presents a follow-up case of Goldenhar Syndrome in a 12-year-old female who had no relevant family history.

## Patient and observation

**Figure 1:**
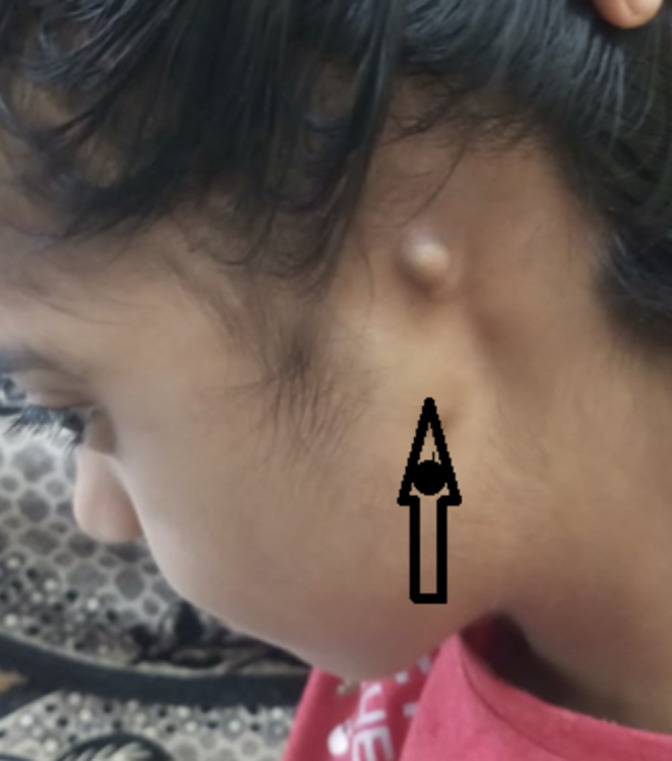
absent ear cartilage and external auditory canal on the left side

A 12-year-old female, who was the firstborn of a non-consanguineous marriage, was diagnosed with anotia on the left side, cyanosis, and facial asymmetry at birth. Figure 1 shows absent ear cartilage and external auditory canal on the left side. She was operated for truncus repair with 12mm aortic homograft and PDA ligation was performed at 2 months of life. She was then put on anti-failure drugs. On examination of the cardiovascular system, S2 had a wide split, and ejection systolic murmur was of intensity 3/6 at the lower-left sterna border. The oxygen saturation (SPO_2_) was 68 % in both the upper extremities. Findings of 2D Echocardiograph suggested of patent ductus arteriosus (PDA), truncus arteriosus type I, ventricular septal defect (VSD), and severe Pulmonary artery hypertension. Ultrasonography disclosed unremarkable study of kidney, ureter and bladder region. The patient grew with normal development and scholastic performance and doing well in school but had restrictions on physical exertion. Moreover, she had moderate growth failure. Upon audiogram, right ear mild conductive hearing loss and left moderately severe conductive hearing loss was found. High-resolution computed tomography (HRCT) of the Petrous Temporal was suggestive of non-visualisation of left external ear (pinna), left auditory canal suggested of agenesis of the left external ear. Both mastoid air cells demonstrated sclerosing and loss of pneumatisation, which was suggestive of bilateral sclerosing mastoiditis. Besides, the course of the left facial nerve was normal.

**Figure 2:**
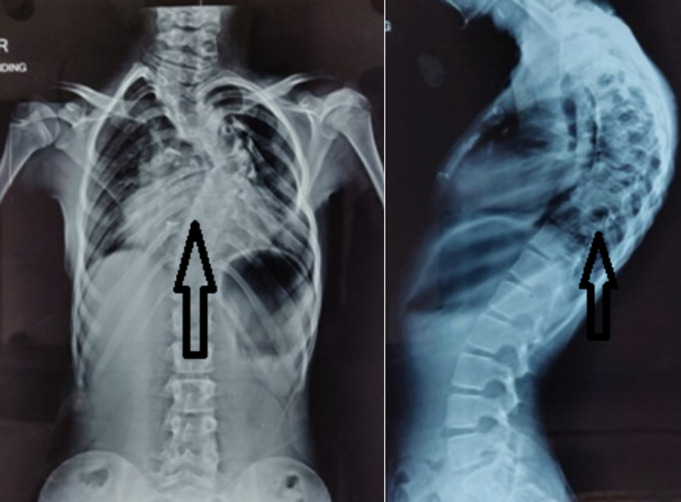
pre-op - X-Ray of the whole spine; (A) standing view shows thoracic curve, uev-d5, lev-d11, apex-d7, cobb- 42 degree; (B) supine side bending view shows proximal thoracic curve, uev- d1, lev -d5, apex-d2, Cobb -40 degree

**Figure 3:**
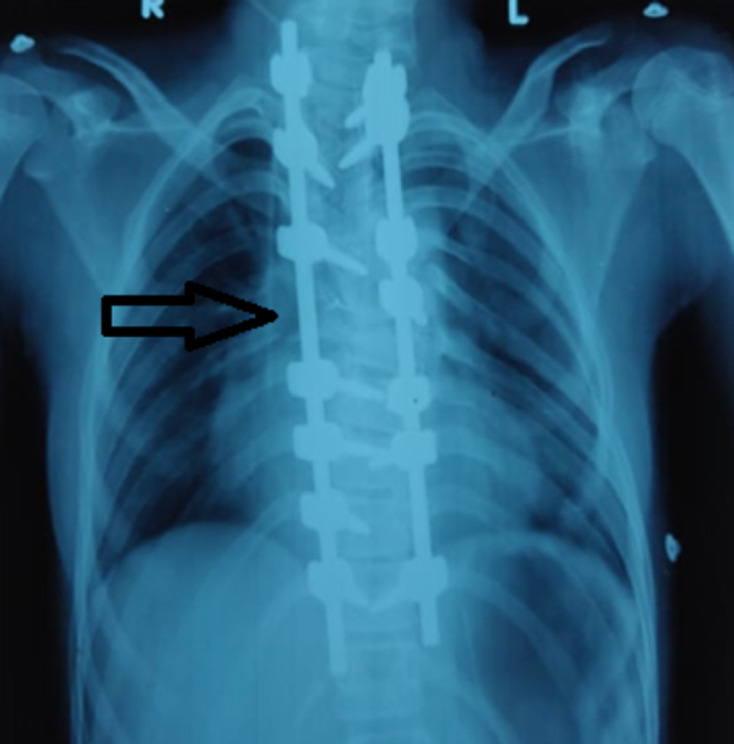
post-op - posterior scoliosis correction with posterior instrumented fusion from (D1 to D11)

Patient started to have a deformity of upper back at the age of 10 years, which was progressive and caused mild left shoulder elevation. She did not present with neurological deficits. Her coronal and sagittal balance was good without truncal imbalance. The X-ray of the spine showed kyphoscoliosis as shown in Figure 2. Both left thoracic curve and right proximal thoracic curve were rigid in finding, with a rib hump of grade 3. Magnetic resonance imaging (MRI) of the whole spine showed the congenital fusion of C2 and C3 vertebrae with the presence of a rudimentary disc in between. C5 and C6 vertebrae showed partial fusion. D1, D2 and D7 level showed the presence of hemi vertebrae, while D5 and D6 showed butterfly vertebrae. The cervicodorsal region had marked kyphoscoliosis. The patient underwent posterior scoliosis correction with posterior instrumented fusion from D1 to D11 for the correction of double thoracic congenital scoliosis with D2 and D7 wedge vertebra (Figure 3). The patient was advised left pinna construction by plastic surgery for the cosmetic purpose to augment symmetry of face although it would not improve hearing ability of left ear. Furthermore, recent cardiac catheterization study showed severe obstruction across pulmonary homograft with grade II Pulmonary Regurgitation and hypertensive dysfunctional right ventricle warranting yet another surgery for homograft replacement. The patient´s parents gave informed consent for reporting herradiography and clinical.

## Discussion

GHS is a rare inherited condition with an uncertain etiology and is identified as a triad of eye dermoids, mandibular hypoplasia and accessory tragic [6]. This condition is characterized by multiple abnormalities that affect the first and second bronchial arches of the first pharyngeal pouch, first brachial cleft, and primordial of the temporal bone [3,7]. This condition develops towards the end of the 7^th^ or 8^th^ week of embryonic life [3]. Bragagnolo *et al*. in 2017 stated that most cases of this syndrome were sporadic with an unknown etiology [8].Their findings revealed repeated chromosomal imbalances that indicated multiple pathogenic pathways involved in this syndrome [8]. GHS accompanies sensor neural hearing impairment, vertebral defects, CNS and renal deformities [9]. Unilateral facial hypoplasia occurs as a facial defect. The most common eye abnormalities include upper eyelid colobomas, subconjunctival dermoids, and epibulbar choristoma [10]. Anomalies of the ears include preauricular skin tags or blind fistulas with aplasia and external auditory meatal Artesia. In the present case, preauricular tags of left pinna were advised for correction by plastic surgery. The heart problems associated with GHS include ventricular septal defects, hypoplasia of the external carotid artery, tetra logy of Fallot with or without a right aortic arch, and tubular hypoplasia of the aortic arch [3]. The renal defects may include an impaired blood supply to the kidney, absence of kidney, double ureter in the kidney and hydronephrosis [3]. Among respiratory system defects, the trachea esophageal fistula is the most prevalent. Besides, they may include incomplete lobulation, hypoplasia or in severe cases, complete agenesis [3]. CNS involvement accompanies with mental retardation. There might be involvement of seventh cranial nerve and unilateral aplasia of the trigeminal nuclei and trigeminal anesthesia. The oral manifestations of GHS include delayed tooth development, cleft lip and palate, hypoplasia of the maxillary and mandibular arches, unilateral tongue hypoplasia, and dentin abnormalities.

Sixty per cent of cases have cervical vertebra unions. Most patients present with Spina Bifida, hemi vertebrae, butterfly, Klippel-Feil anomaly [3]. Besides, GHS may include unilateral micrognathia, unilateral mandibular hypoplasia, epibulbar dermoids cysts or vertebral malformations. According to Caron *et al*. spine anomalies are significantly more common in patients with a more severe mandibular hypoplasia and bilateral anomalies than in patients with unilateral anomalies [11]. In our patient, the correction of double thoracic congenital scoliosis with D2 and D7 wedge vertebra was performed by posterior scoliosis correction posterior instrumented fusion from D1 to D11. Since there are no genetic or chromosomal tests for the diagnosis of GHS, a specialist diagnoses the syndrome by identifying GHS symptoms. When identified, the child will usually need additional tests, such as vision and hearing tests, and kidney or heart exams. At a minimum, two of the following findings for the diagnosis of GHS should be present, which are unilateral micrognathia, epibulbardermoids cysts, unilateral mandibular hypoplasia, or vertebral malformations [3].GHS treatment is dependent upon age and systemic relationships. In uncomplicated cases, the prognosis of the disease is good [3].

## Conclusion

In conclusion, Goldenhar syndrome patients can have multiple congenital abnormalities and must be thoroughly examined with a multidisciplinary approach to carry out appropriate therapeutic planning and periodic evaluations in monitoring the child's growth and development. It can interfere with many aspects of patient life. At present, the patient is in medical care and observation for any possible problem.
